# Supplementary material for: Structure of a step II catalytically activated spliceosome from Chlamydomonas reinhardtii
Source: EMBO J. 2024 Oct 16;44(4):975–90. doi: 10.1038/s44318-024-00274-3 (PMC11833078; doi:10.1038/s44318-024-00274-3)
Supplement: Supplementary file 1 — Appendix [file 44318_2024_274_MOESM1_ESM.pdf]

Appendix

Structure of a Step II Catalytically Activated Spliceosome from *Chlamydomonas reinhardtii*

Yichen Lu, Ke Lian, Xiechao Zhan

Table of contents:

Appendix Figure S1 Page2
Appendix Figure S2 Page3
Appendix Figure S3 Page4
Appendix Figure S4 Page5
Appendix Figure S5 Page6
Appendix Table S1 Page7
Appendix Table S2 Page8
Appendix Table S3 Page10
Appendix References Page11

Appendix Figure S1

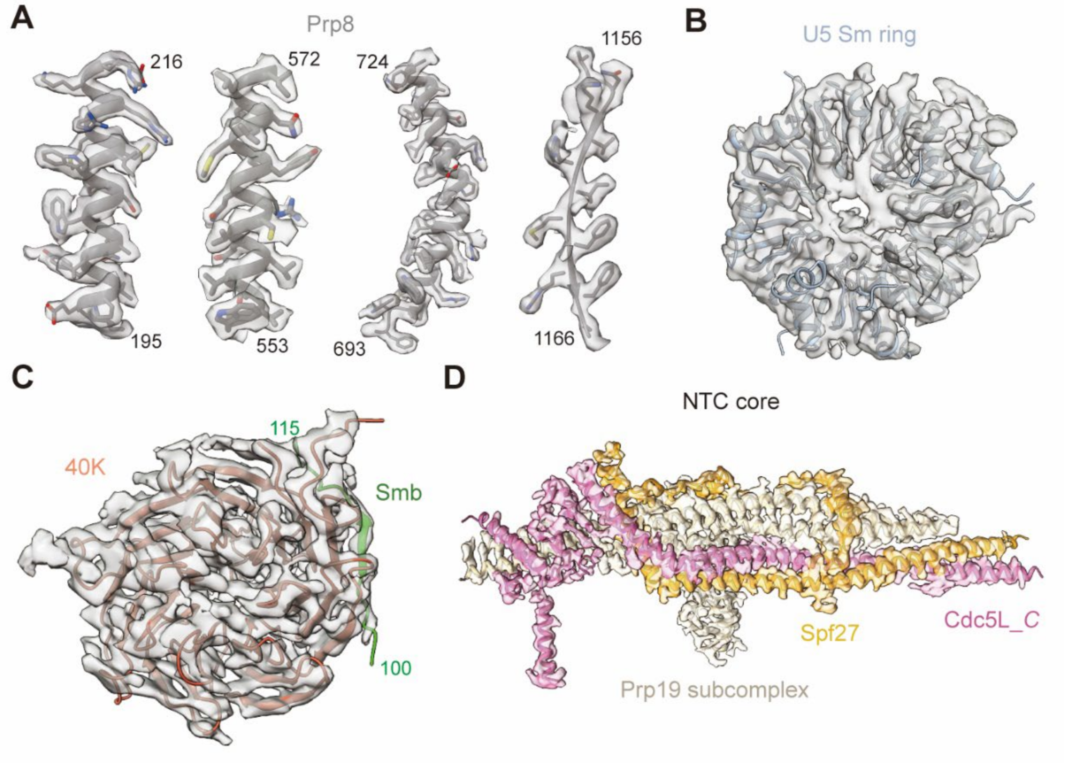

Appendix Figure S1. Representative EM maps of protein components in the *Chlamydomonas* C* complex.

(**A**) The representative EM maps of Prp8. (**B**) The EM map of U5 Sm ring. (**C**) The EM maps of U5-40K and the C-terminus of Smb. (**D**) The EM maps of the NTC core.

Appendix Figure S2

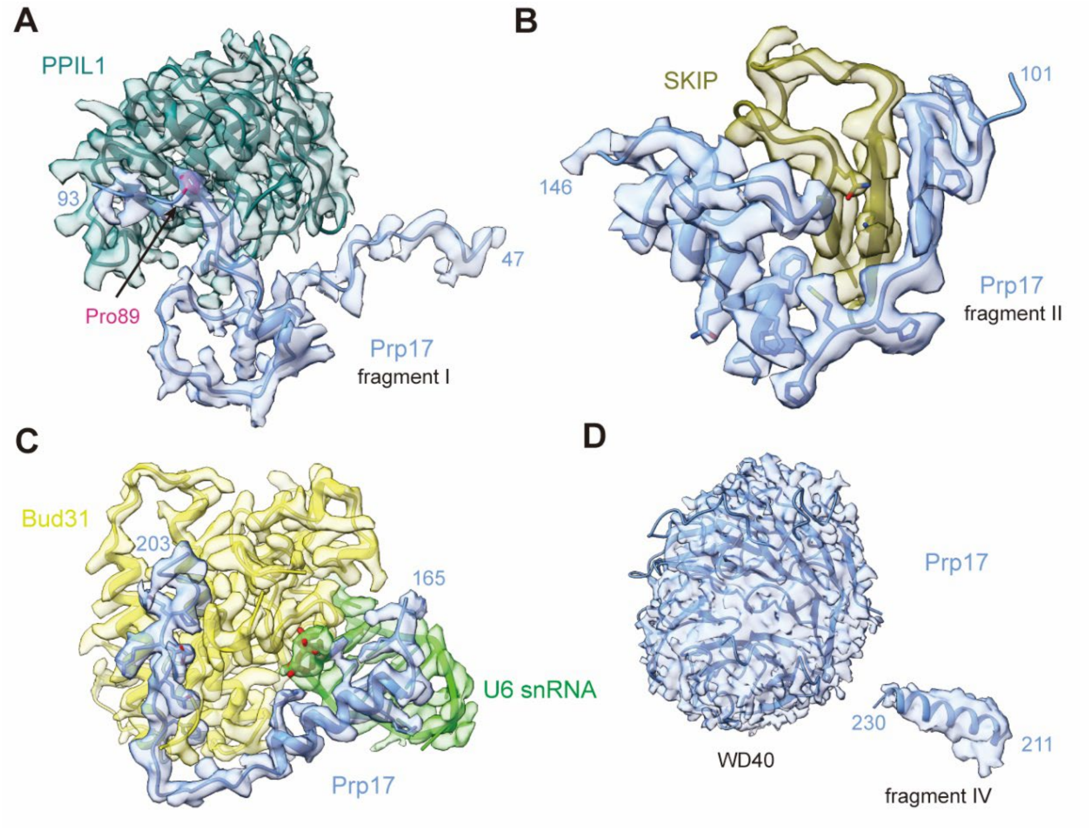

Appendix Figure S2. The EM maps of protein components around Prp17.
(**A**) The EM maps of PPIL1 and the fragment I of Prp17. (**B**) The EM maps of SKIP and the fragment II of Prp17. (**C**) The EM maps of the interface among the 5'-SL of U6 snRNA, Bud31, and the fragment III of Prp17. (**D**) The EM maps of the fragment IV and WD40 domain of Prp17.

Appendix Figure S3

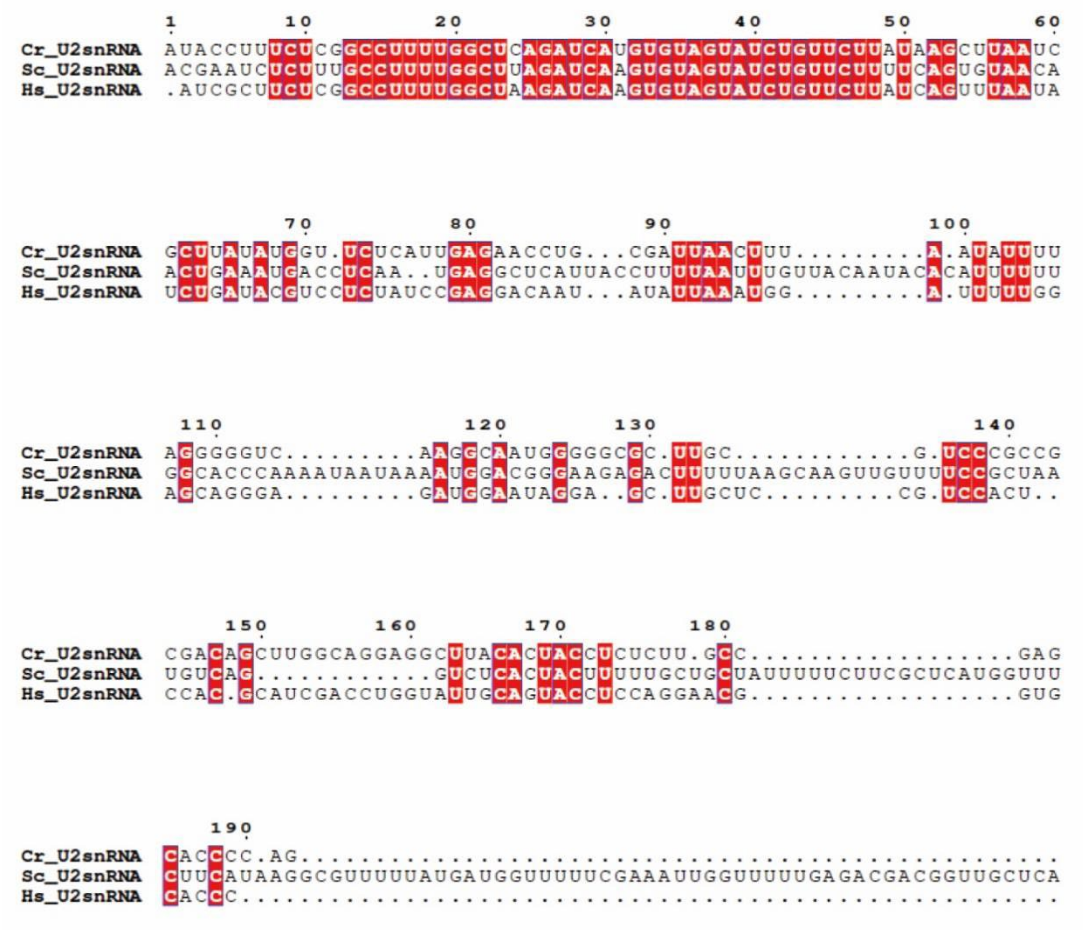

Appendix Figure S3. The sequence alignment of *Chlamydomonas* U2 snRNA with its orthologs from *S. cerevisiae* (Sc) and human (Hs).

Conserved nucleotides are boxed. Invariant sequences are highlighted in red background. Sequence alignment was performed using the Clustal Omega server[1] and the figure was generated using the ESPript server[2].

Appendix Figure S4

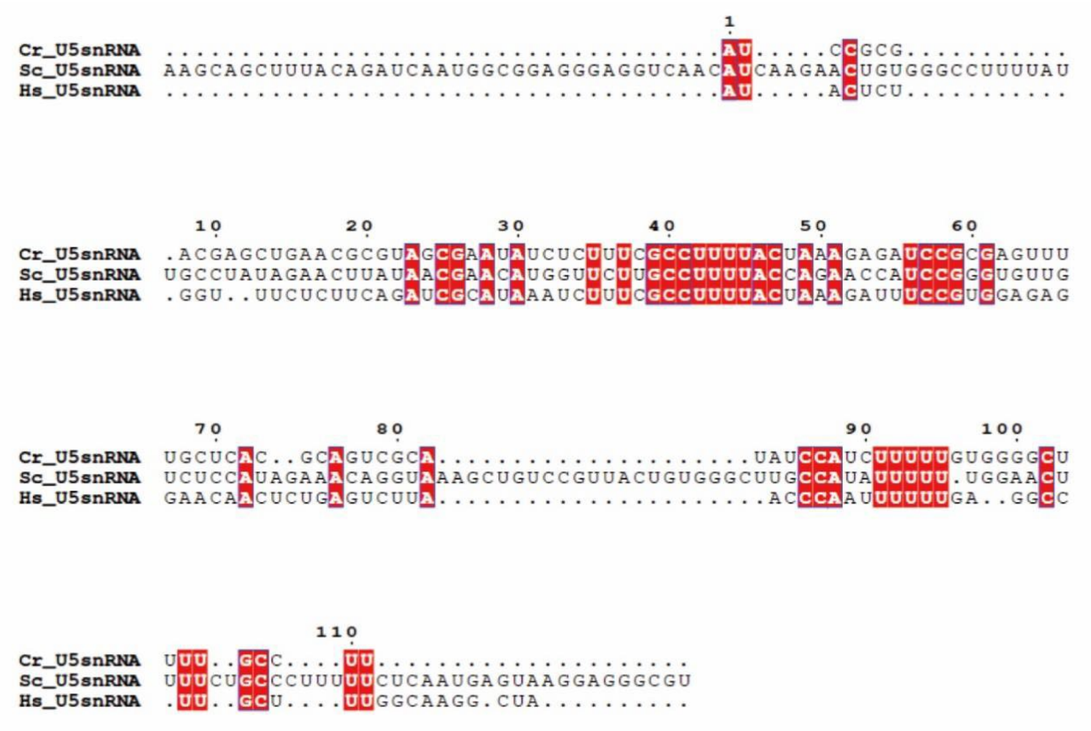

Appendix Figure S4. The sequence alignment of *Chlamydomonas* U5 snRNA with its orthologs from *S. cerevisiae* (Sc) and human (Hs).

Conserved nucleotides are boxed. Invariant sequences are highlighted in red background. Sequence alignment was performed using the Clustal Omega server[1] and the figure was generated using the ESPript server[2].

Appendix Figure S5

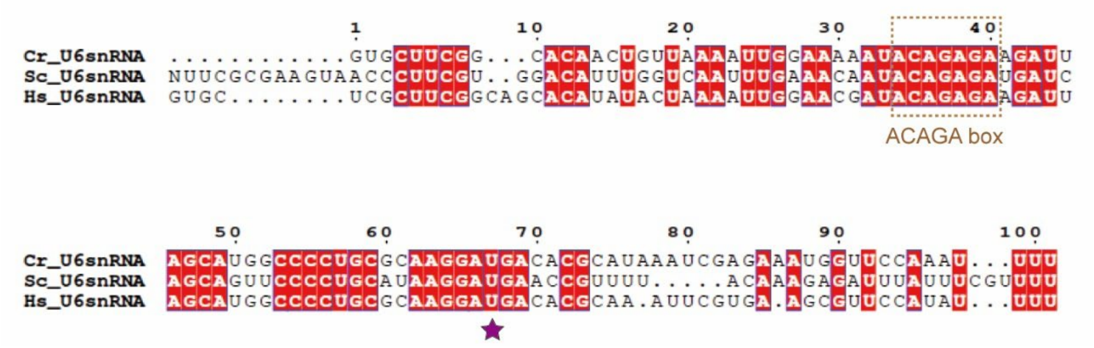

Appendix Figure S5. The sequence alignment of *Chlamydomonas* U6 snRNA with its orthologs from *S. cerevisiae* (Sc) and human (Hs).

Conserved nucleotides are boxed. Invariant sequences are highlighted in red background. The key nucleotide U67 is labeled with a purple star. Sequence alignment was performed using the Clustal Omega server[1] and the figure was generated using the ESPript server[2].

Appendix Table S1. Statistics of 3D reconstructions and model refinement.

	Cdc5L-Flag dataset	Prp19-Flag dataset
**Data collection**		
EM equipment	Titan Krios G4	Titan Krios G3i
Voltage (kV)	300	300
Detector	Falcon 4i	K3
Magnification	130,000	81,000
Pixel size (Å)	0.92	1.077
Electron dose (e-/Å^2)	50	50
Defocus range (μm)	-1.0 ~ -1.5	-1.4 ~ -1.8
**Reconstruction**		
Software	RELION 3.0/cryoSPARC	
Symmetry	C1	
Number of particles	518,369	521,250
Final masked resolution (Å)	2.6	2.6
FSC threshold	0.143	
Map sharpening B-factor (Å^2)	-66.9	-87.4
EMDB code	EMD-38362	EMD-38366
**Model building**		
Software	Coot-0.8.9/Chimera	
Refinement	Phenix	
Protein residues	9,683	
RNA nucleotides	258	
B factors (Å^2)	24.5	
PDB code	8XI2	
**Validation**		
R.m.s deviations		
Bonds length (Å)	0.012	
Bonds Angle (°)	0.996	
Ramachandran plot statistics (%)		
Favored	93.33	
Outlier	0.20	
Clashscore	21.1	
CaBLAM outliers (%)	2.6	
MolProbity score	3.1	

Appendix Table S2. Summary of modeled components in the *Chlamydomonas* C* complex.

Sub-complexes	Molecule *Chlamydomonas*/**human**/*S. cerevisiae*	Gene ID	UniProt ID	Length	Modeled region	PDB template	Modelling method	Chain ID
U5 snRNP	U5 snRNA	GenBank: X67000.1	-	111 nt	7-80<br>87-103	-	HM	B
	Prp8/PRP8/Prp8	Cre01.g016050	A0A2K3E5Q8	2398	84-342<br>355-412<br>447-2079	6ID1	HM	A
	Snu114/SNU114/Snu114	Cre17.g737250	A8JCA8	989	66-962	AF predicted	RD	C
	U5-40K/U5-40K/-	Cre06.g255400	A0A2K3DMA3	362	45-362	AF predicted	RD	E
	SmB/SmB/SmB	Cre14.g611150	A0A2K3CX88	265	7-89	AF predicted	RD	b
	SmD1/SmD1/SmD1	Cre16.g665400	A0A2K3CTT9	114	1-88	AF predicted	RD	c
	SmD2/SmD2/SmD2	Cre10.g45345	A8I074	110	11-110	AF predicted	RD	d
	SmD3/SmD3/SmD3	Cre10.g455300	A8I0E2	127	5-94	AF predicted	RD	a
	SmE/SmE/SmE	Cre08.g385200	A8IYZ2	87	8-87	AF predicted	RD	e
	SmF/SmF/SmF	Cre05.g241300	A8J834	85	7-77	AF predicted	RD	f
	SmG/SmG/SmG	Cre13.g566050	A8HRS8	77	7-71	AF predicted	RD	g
U6 snRNP	U6 snRNA	GenBank: X71486.1	-	101 nt	1-92	5XJC	HM	F
Pre-mRNA	Intron	-	-	/	1-23<br>90-101	5XJC	DM	3
	5'-exon	-	-	/	-7~-1	5XJC	DM	5
U2 snRNP	U2 snRNA	GenBank: X71483.1	-	192 nt	1-40	5XJC	HM	H
PRP19/NTC complex	Prp19/PRP19/Prp19	Cre02.g073650	A8I9S6	503	q: 1-118<br>r: 1-131<br>s: 67-136<br>t: 63-136	AF predicted	RD	q,r,s,t
	Spf27/SPF27/Snt309	Cre17.g697350	A0A2K3CNP4	303	26-235	AF predicted	RD	K
	Cdc5L/CDC5L/Cef1	Cre03.g197350	A0A2K3DYR4	833	7-239<br>541-833	AF predicted	RD	L
	Cwc15/AD-002/Cwc15	Cre24.g755447	A0A2K3CN43	235	9-78<br>194-235	AF predicted	RD	P
	PLRG1/PLRG1/Prp46	Cre10.g446900	A0A2K3DAW8	518	175-518	AF predicted	RD	T
NTC Related Complex (NTR)	Syf2/SYF2/Syf2	Cre01.g037400	A0A2K3E769	563	441-510<br>529-543	AF predicted	RD	M
	Syf3/SYF3/Clf1	Cre14.g621000	A0A2K3CXZ4	835	16-41<br>47-643	AF predicted	RD	J
	Bud31/BUD31/Bud31	Cre10.g442400	A0A2K3DAL7	233	4-179	AF predicted	RD	N
	RBM22/RBM22/Cwc2, Ecm2	Cre02.g075650	A0A2K3E050	417	18-124<br>144-303	AF predicted	RD	O
	SKIP/SKIP/Prp45	Cre01.g051100	A0A2K3E831	684	35-265<br>274-302	AF predicted	RD	R
	PPIL1/PPIL1/Cpr2	Cre12.g499400	A8JGI0	157	3-157	AF predicted	RD	S
Intron Binding Proteins (IBP)	Syf1/SYF1/Syf1	Cre12.g513500	A0A2K3D3K4	925	69-761	AF predicted	RD	I
	Aquarius/AQR/-	Cre06.g300750	A0A2K3DR41	1844	118-1777	5XJC	HM	Q

Splicing Factors	Cwc21/SRM300/Cwc21	Cre09.g409200	A0A2K3DFH0	721	1-26	AF predicted	RD	U
	Cwc22/CWC22/Cwc22	Cre06.g259800	A0A2K3DML4	928	650-835	AF predicted	RD	V
	Prp17/PRP17/Prp17	Cre04.g226450	A0A2K3DUN6	576	47-146<br>165-203<br>211-230<br>237-576	AF predicted	RD	W

Under the column labeled "Molecule", proteins from *Chlamydomonas*, human, and *S. cerevisiae* are colored black, red, and blue, respectively. Under the column labeled "PDB template", AF predicted means AlphaFold-predicted structure. Under the column labeled "Modelling method", RD stands for rigid docking and manual adjustment; HM stands for homology modelling; DM stands for *de novo* modelling.

Appendix Table S3. Summary of components comparison among the *Chlamydomonas*, human and *S. cerevisiae* C* complexes.

Sub-complexes	Molecule	*Chlamydomonas* (this study)	Human (PDB: 7W5B)	*S. cerevisiae* (PDB: 5WSG)
U5 snRNP	U5 snRNA	√	√	√
	Prp8	√	√	√
	Snu114	√	√	√
	U5-40K	√	√	Not in genome
	U5 Sm ring	√	√	√
	Brr2	Not observed	√	
U6 snRNP	U6 snRNA	√	√	√
Pre-mRNA	Intron	√	√	√
	5'-exon	√	√	√
U2 snRNP	U2 snRNA	√	√	√
	U2-A'	Not observed	√	√
	U2-B"	Not observed	√	√
	U2 Sm ring	Not observed	√	√
PRP19/NTC complex	Prp19	√	√	√
	Spf27	√	√	√
	Cdc5L	√	√	√
	Cwc15	√	√	√
	PLRG1	√	√	√
NTC Related Complex (NTR)	Syf2	√	√	√
	Syf3	√	√	√
	Bud31	√	√	√
	RBM22	√	√	(Ecm2, Cwc2) √
	Skip	√	√	√
	PPIL1	√	√	Not observed
Intron Binding Proteins (IBP)	Syf1	√	√	√
	Aquarius	√	√	Not in genome
	PPIE	Not observed	√	Not in genome
Splicing Factors	Cwc21	√	√	√
	Cwc22	√	√	√
	Prp17	√	√	√
	Prp22	Not observed	√	√
	SLU7	Not observed	√	(Prp18) √
	FAM32A	Not observed	√	Not in genome
	PRKRIP1	Not observed	√	Not in genome
	Cactin	Not observed	√	Not in genome
	NKAP	Not observed	√	Not in genome
EJC complex	/	Not observed	√	Not in genome

Appendix References

1 Madeira, F. *et al.* Search and sequence analysis tools services from EMBL-EBI in 2022. *Nucleic acids research* **50**, W276-W279, doi:10.1093/nar/gkac240 (2022).

2 Gouet, P., Courcelle, E., Stuart, D. I. & Metoz, F. ESPript: analysis of multiple sequence alignments in PostScript. *Bioinformatics* **15**, 305-308, doi:10.1093/bioinformatics/15.4.305 (1999).
