# Supplementary material for: Structure of a step II catalytically activated spliceosome from Chlamydomonas reinhardtii
Source: EMBO J. 2024 Oct 16;44(4):975–90. doi: 10.1038/s44318-024-00274-3 (PMC11833078; doi:10.1038/s44318-024-00274-3)
Supplement: Supplementary file 3 — Expanded View Figures [file 44318_2024_274_MOESM3_ESM.pdf]

Expanded View Figures

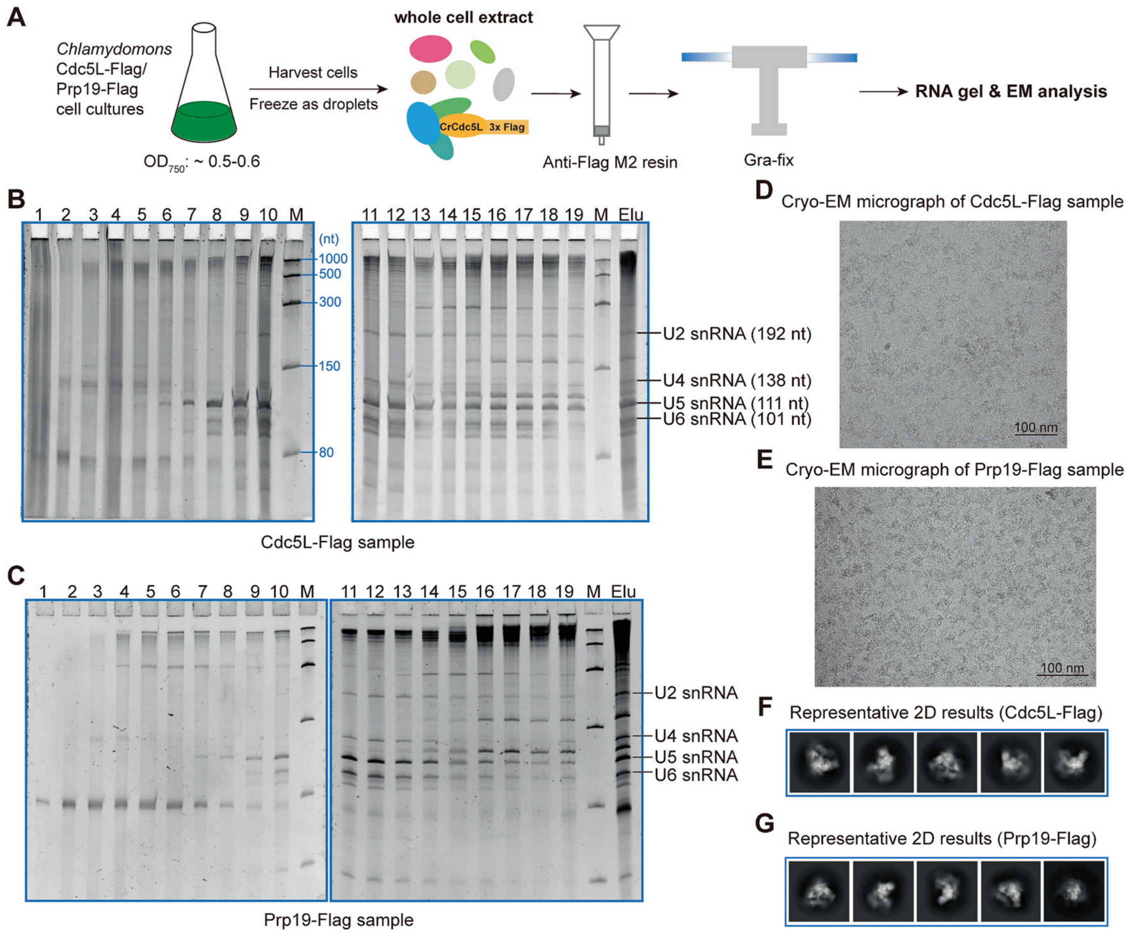

Figure EV1. Purification and analysis of *Chlamydomonas* spliceosomal complexes.

(**A**) A simplified schematic diagram for the isolation of *Chlamydomonas* spliceosomal complexes. (**B**, **C**) The putative spliceosomal complexes were analyzed after glycerol gradient centrifugation with chemical crosslinking using urea-PAGE gels from the *Chlamydomonas* Cdc5L-Flag (**B**) and Prp19-Flag (**C**) strains. (**D**, **E**) Representative cryo-EM micrographs of the *Chlamydomonas* spliceosomal complexes from the Cdc5L-Flag (**D**) and Prp19-Flag (**E**) strains. Scale bar, 100 nm. (**F**, **G**) Representative 2D average results of the *Chlamydomonas* spliceosomal complexes from the Cdc5L-Flag (**F**) and Prp19-Flag (**G**) datasets.

 © The Author(s)

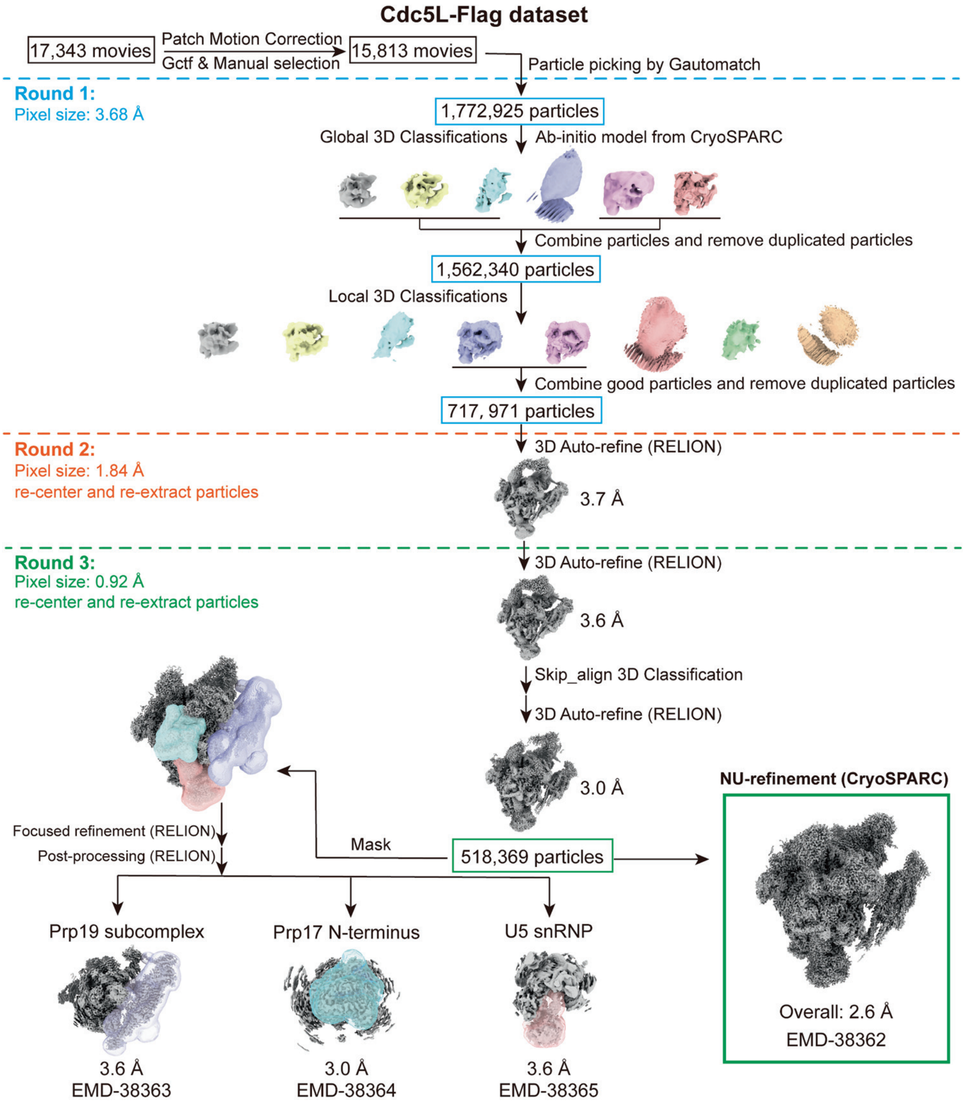

Figure EV2. A flow chart of cryo-EM data processing for the *Chlamydomonas* C* complex from the Cdc5L-Flag dataset.

All processing steps were carried out in RELION 3.0 and cryoSPARC. Please refer to Methods for details.

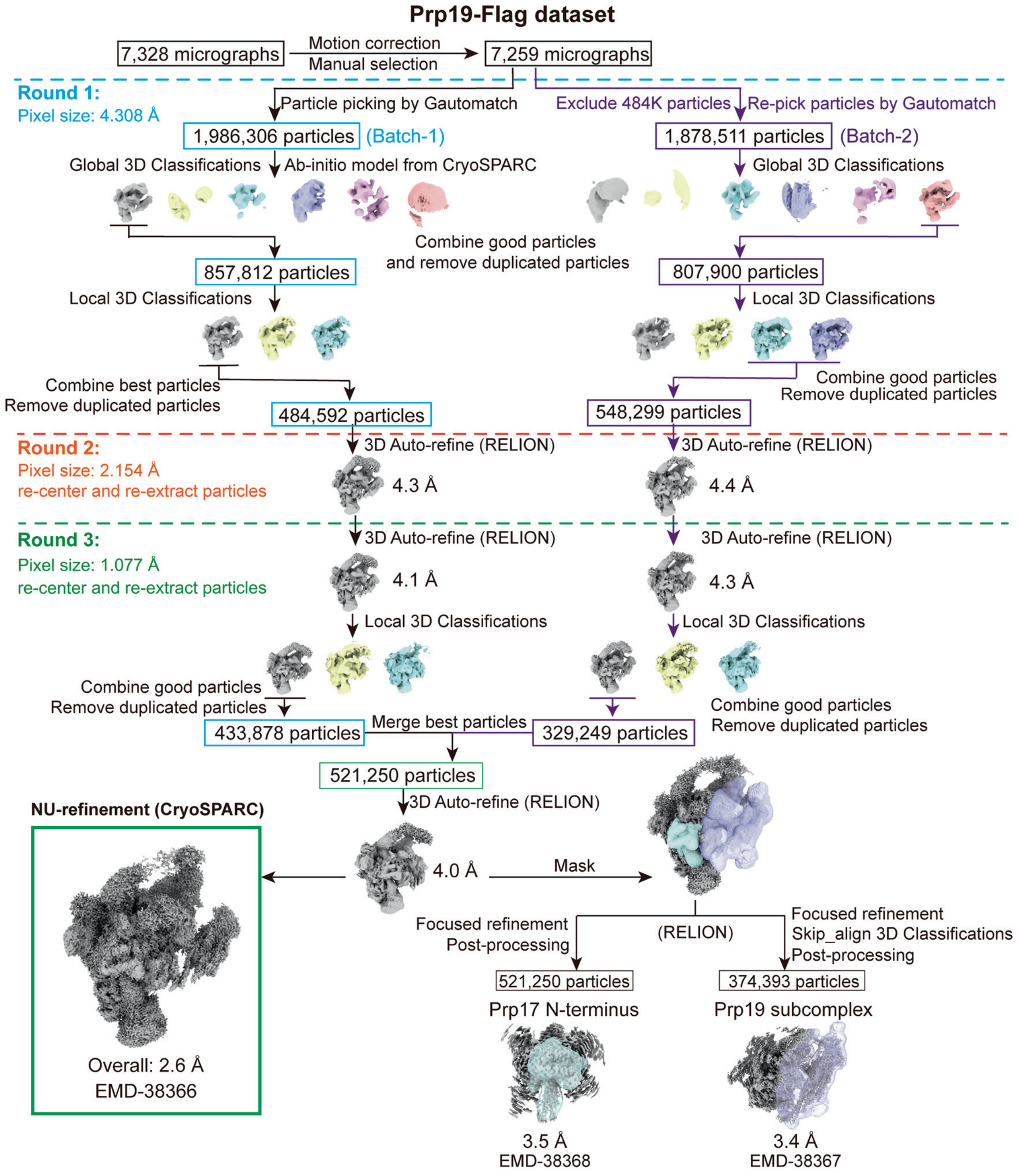

Figure EV3. A flow chart of cryo-EM data processing for the *Chlamydomonas* C* complex from the Prp19-Flag dataset.

All processing steps were carried out in RELION 3.0 and cryoSPARC. Please refer to Methods for details.

 © The Author(s)

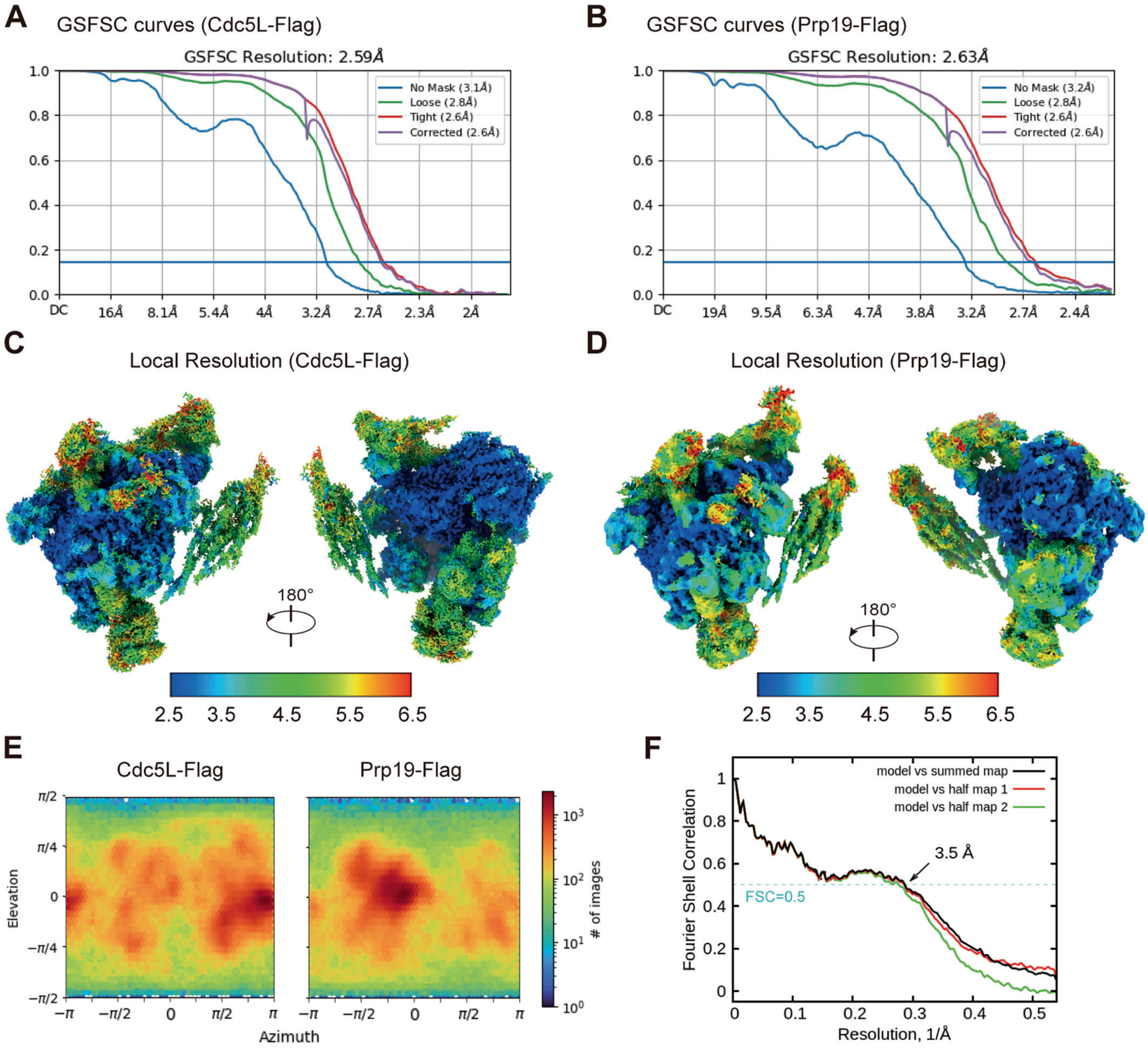

Figure EV4. Cryo-EM reconstruction of the *Chlamydomonas* C* complex.

(**A**, **B**) Shown here are the FSC curves for the final refinement in cryoSPARC. The average resolutions have been both achieved at 2.6 Å for the *Chlamydomonas* C* complex using the Cdc5L-Flag (**A**) and Prp19-Flag (**B**) datasets. (**C**, **D**) Shown here are the local resolutions color-coded for different regions from the reconstructions using the Cdc5L-Flag (**C**) and Prp19-Flag (**D**) datasets. (**E**) Shown here are the angular distributions of the particles used for the final reconstructions in cryoSPARC for the Cdc5L-Flag (left panel) and Prp19-Flag (right panel) datasets. (**F**) The FSC curves for the cross-validation between the model and the cryo-EM maps of the *Chlamydomonas* C* complex. Shown here are the FSC curves between the final refined atomic model and the reconstruction from all particles (black), between the model refined in the reconstruction from only half of the particles and the reconstruction from that same half (red), and between that same model and the reconstruction from the other half of the particles (green).

© The Author(s)

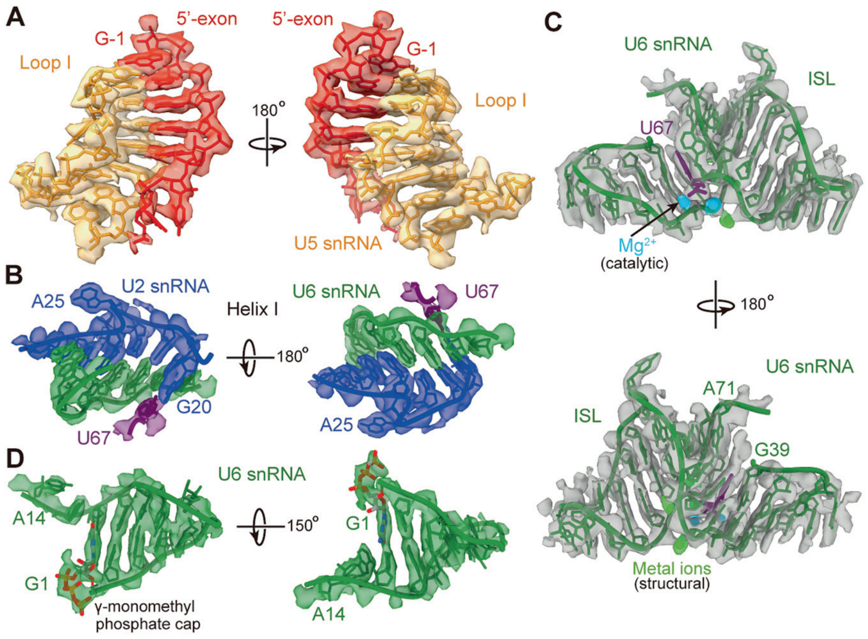

Figure EV5. Representative EM maps of the RNA elements.

(**A**) The EM maps of the 5′-exon and loop I of U5 snRNA. Two related views are shown. (**B**) The EM maps of the Helix I. Two related views are shown. (**C**) The EM maps of the ISL of U6 snRNA. Two catalytic metal ions are coordinated by the key nucleotide U67. Two related views are shown. (**D**) The EM maps of the 5′-SL of U6 snRNA. Two related views are shown.

 © The Author(s)
